# Monthly Summary

**Published:** 1881-05

**Authors:** 


### MONTHLY SUMMARY.

*Holland's Dental Law.*—[Translated by George H. Perine, D. D. S., New York.]—Below is a translation of the Dutch law pertaining to the practice of dental surgery in Holland. In the Netherlands dentistry for years was restricted by a separate law. In the year 1865 it was decided to make the law uniform, and those wishing to practice dental surgery were compelled to study medicine; the degree of Doctor of Medicine entitled them to practice dental surgery without further study or examination. But few persons, however, directed exclusive attention to dentistry in Holland up to 1878, when in December of that year a law was passed regulating the conditions for obtaining the degree to practice medicine, midwifery, dentistry, etc. The new law pertaining to dentistry provides as follows:

*Article I.*—For the degree of dentist (Trandmuster—licentiate to practice dentistry), by which is understood local treatment of the diseases to which the teeth, alveolar process, and gums are liable.

*Article II.*—This degree is obtained after having passed the practical examination successfully,—that is, satisfactory proofs are required of the knowledge of operative dentistry and the placing of artificial dentures.

*Article III.*—To the practical examination those only are admitted who have passed their theoretical examination on dentistry successfully.

*Article IV.*—The theoretical examination comprises the anatomy of the teeth, alveolar process, and gums; their physiology, their hygiene, pathology, and therapeutics, in which is included the knowledge of diagnosing the diseases of the teeth, alveolar process, and gums, whether arising locally or remo e (constitutional,) pharmacodynamie, and the art of prescribing as is necessary,—prescribing local medicines for the diseases o the above-named parts.

*Article V.*—The medical universities of the Netherlands are qualified (have the right) to examine on theoretic dentistry, and to give a certificate to those who have successfully passed the same.

*Article VI.*—The amount of five-and-twenty (25) guilders must be paid to the president of the faculty by the applicant before submitting to the examination. In case of being unsuccessful, no second payment is required at the second application.

*Article VII.*—A diploma will be given when the examinations have been passed satisfactorily, and the amount of twenty-five guilders has been paid to the president of the committee, which entitles the possessor to style himself physician, dentist, etc.

*Article VIII.*—All those having passed their examination successfully must, before being admitted, take in the presence of the president of the examining board the following oath (or promise:) I swear (or promise) that I will practice dentistry according to the legally fixed stipulations, and to the best of my knowledge and ability; that I never in my practice will disclose to any one what in secret is trusted to me, or come to my knowledge, except when my testimony is required as witness or expert by the court, or I am legally obliged to give information.

So help me God Almighty (that I promise).

Resolutions passed February 12, 1879 :

*Article I.*—Two periods will be appointed in the civil year for making the examinations,—the first lasting six weeks in the first eight months, and the last for four weeks during the remaining four months. Day and hour will be appointed by the president of the faculty, or by the member who fills his place.

*Article II.*—The faculty convenes with no less than three members present, and examine but one at a time. The success of the candidate must be decided by a majority vote. If a division of the vote is for the rejection of the candidate, this rejection is for one year.

*Article* III.—The examination is made in the Dutch language, unless the faculty (board) consents to the use of a foreign language at the request of the candidate. All examinations are public unless the board otherwise decides.

*Article IV.*—A dated certificate, and written in the Dutch language, signed by the president, or *loco* president, will be given to him who passes his examination successfully.

*Article V.*—The day and hour for examination are fixed by the president of the board, or *loco* president, which is communicated by the secretary to the parties concerned.

(Signed) WILLEM,
Minister of Interior.
(Signed) KAPPEYNE.

GRAVENHAGE, 12th February, 1879.—*Cosmos.*

---

*Extraction of the Teeth followed by Insanity.*—On the morning of April 20th, 1880, I was consulted by Frank McVey, aged 23 years, in reference to the extraction of the roots of the superior six year and twelve year old molars, and second bicusped. The patient, a well nourished young man, was suffering from ordinary chronic compound alveola abscess (from more than one root) with opening into the antrum highmore. We advised the extraction of all the roots.

In a large dental practice of more than eleven years, I never saw before, a patient exhibit more terror from teeth extraction; and before operating, prescribed in two doses, three ounces of good apple brandy. The liquor appeared to allay the excessive nervousness and fear of the patient, and in a few minutes proceeded to operate, and extracted without trouble, the roots of the above mentioned teeth, three of which (roots) were connected with encysted abscesses. I thoroughly syringed the antrum highmore with a one to twenty solution of carbolic acid, and ordered it to be continued for five days.

The patient was not particularly affected by the liquor, nor did he appear to have received a nervous shock, but worked the balance of the day on the Bound Brook Railroad.

The *next* day, however, cerebral disorder was manifested, the young man acted strange, had an anxious careworn look, was very dull, complained rather incoherent of pain in the head, and objected to go to work.

The family physician, Dr. Tompkins, of Harlingen, N. J., was called in and diagnosed *congestion* of the brain, and prescribed appropriate remedies. After one month's treatment, the patient was no better, the wounds in the gum were healed in the usual time; no discharge whatever from antrum highmore after the third day.

December 20th, 1880; eight months have elapsed since the teeth were extracted. I was informed to-day by a brother of the unfortunate young man, that he is no better, and his parents have determined to remove him to the Trenton Asylum.

Insanity in this case presents itself as imbecility. A most important point in the etiology of this case, is the sensitive temperament of the unfortunate youth, no other hypochondriac or *functional* neurosis.

There is no mania or dementia, hysteria or epilepsy, no threatening menaces, or atonic agitation. The entire symptomology is summed up in the one expression, imbecility. I have examined the heredity of the entire family, but found no acute evidence of insanity in a record of many years

REMARKS.—The father of the young man, (a well to do farmer,) is a moderate drinker, the mother is hysterical, and suffering, with nearly every member of the family, Post-Nasal Catarrh of a very offensive character.

After many engagements and much trouble, I succeeded in extracting the mother's upper teeth, which were in a state of complete disintegration, and since the insanity of the son, have extracted the teeth of the oldest daughter, in which case, ether was administered by Dr. Abraham Moses, of Griggstown, N. J., and sixteen worthless teeth extracted at one sitting, no pain during the operation, or bad after effects. Both mother and daughter are wearing artificial teeth with comfort.—*Edward H. Bowne, Rock Hill, N. J.*

QUERY.—Does the extraction of the teeth bear on the case, or is the *constitutional* disease of the family, Post-Nasal Catarrh, *a* factor in the case?—*Dental Luminary.*

---

*To Prevent the Clouding of Mirrors by Moisture.*—The clouding of a looking glass in the vapor-laden atmosphere of the bath-room, or from the breath, may be simply an annoyance to one who is shaving, but in the use of many instruments in operative surgery, the several forms of speculums, the laryngoscope, etc., and in astronomical observations, the formation of dew on the lenses of telescopes, are very serious impediments to the operator and observer. The trouble however, may be simply and effectually obviated, says the *Manufacturer and Builder*, by passing lightly over the surface of the glass or speculum a cloth or sponge slightly moistened with glycerine. Glycerine has considerable avidity for moisture, and in consequence the vesicles of water vapor contained in the expired breath or in the moist atmosphere of the bath-room, in touching this surface are instantly dissolved, so that no cloud is formed. If the reader will try this simple receipt, he will find himself relieved of the annoyance alluded to, and will be able to shave in peace. We can particularly recommend this simple and effective remedy against the tarnishing of mirror surfaces by moisture to the medical profession, as vastly more practical and better than the practice now in vogue of dipping them in tepid water or haeting slightly over a lamp before using their instruments, as by the simple use of glycerine in the manner we have described, there will be no fear of giving the patient an unpleasant or painful sensation by accidental overheating of the mirror when applied to sensitive or inflamed parts; and the glycerine keeps the mirror from tarnishing long enough to suffice for half a dozen operations, whereas in the old way, the mirror, by radiation and conduction, soon loses its acquired heat and becomes clouded again, often before the operation or observation is finished. We would say, in conclusion, that this question points the moral that trifling things are not to be despised.—*Druggists Circular.*

---

*A Case of Fracture of the Teeth Producing Severe Symptoms.*—Under the care of Dr. Wilks. Communicated by W. Hale White, M. B., Lond., House Physician.

E. M—, a boy, æt 13, admitted into Stephen Ward on May 17th, 1880. Three days before admission had a quarrel with another boy, who struck him in the mouth with a scoop, breaking off the upper half of his two central incisors, and the anterior surfaces and upper halves of his two lateral incisors, the pulp being quite exposed. Was quite well up to this date. He cried a little after the accident. but went on with his work. He did not swallow the fragments of teeth. On the same evenhe had diarrhœa, his bowels being opened four times. Next day he had pain in the head and diarrhœa, the bowels acting six times. On the day after he had swimming in the head, shivering, pain in the abdomen and teeth; the bowels acted nine times. On the day of admission his bowels had been opened five times by 10 A. M., and had vomited twice. When first seen he had a hot, dry skin, and temperature 100:2°, pulse 120, together with pain in the abdomen. The tongue was furred and the lips tender, but without sore. He is thirsty, and all the organs of the body but the teeth are normal. The four lower incisors are broken off about a third of their height from the gum. The red pulp is exposed; it does not bleed, but is excessively tender. Put on milk diet. A day or two after admission the temperature fell to normal, the diarrhœa stopped, and all symptoms of fever passed off. Four days after admission, the pupils were extirpated, under chloroform, by Mr. Moon, and the cavities plugged with cotton-wood soaked in carbolic acid. This was re-applied six days afterwards, and four days after that the cavities were stopped with gutta-percha and the boy went out well.—*Med. Times and Gazette.*

---

*Alcohol a Stimulant or Narcotic.*—Alcohol, says Dr. Wilks, in the *Monthly Magazine of Pharmacy*, is stated to be a stimulant. If a man is jaded and tired, it affords a temporary support; a little later he is depressed, the stimulant lasting only a short time. It produces dilatation of the vessels and warmth of the surface, but at the expense of internal heat. The fact is, alcohol is not a stimulant at all, *but a depressant and a narcotic.* If the name were changed we should get a proper notion of its character, and Dr. Wilks believes that such a change would tend more than anything else to make people cautious in its imbibition. Alcohol is taken for the same reason as chloral or opium in other countries, and if regarded as a narcotic, the consequences of its use would be better understood. It has a sedative effect, and is therefore, useful in severe neuralgia when chloral and opium fail. It benumbs the sense of touch, as well as that of sight and taste; but if it were a stimulant, then sense of taste ought under its influence to become more refined.—*Druggists Circular.*

